# The orthology of HLA-E and H2-Qa1 is hidden by their concerted evolution with other MHC class I molecules

**DOI:** 10.1186/1745-6150-1-2

**Published:** 2006-01-31

**Authors:** Etienne Joly, Virginie Rouillon

**Affiliations:** 1 Equipe de Neuro-Immuno-Génétique Moléculaire, IPBS, UMR CNRS 5089, 205 route de Narbonne, 31077 Toulouse Cedex, France

## Abstract

**Background:**

Whether MHC molecules undergo concerted evolution or not has been the subject of a long-standing debate.

**Results:**

By comparing sequences of eight functional homologues of HLA-E from primates and rodents with those of MHC class Ia molecules from the same eight species, we find that different portions of MHC class I molecules undergo different patterns of evolution. By focusing our analyses sequentially on these various portions, we have obtained clear evidence for concerted evolution of MHC class I molecules, suggesting the occurrence of extensive interallelic and intergenic exchanges. Intra-species homogenisation of sequences is particularly noticeable at the level of exon 4, which codes for the α3 domain, but our results suggest that homogenisation also concerns certain residues of the α1–α2 codomain that lie outside the antigen recognition site.

**Conclusion:**

A model is presented in which Darwinian selective pressures due to pathogens could, at the same time, favour diversification of MHC class Ia molecules and promote concerted evolution of separate loci by spreading advantageous motifs arising by mutations in individual MHC molecules to other alleles and to other loci of the MHC region. This would also allow MHC molecules to co-evolve with the proteins with which they interact to fulfil their functions of antigen presentation and regulation of NK cell activity. One of the raisons d'être of the MHC may therefore be to favour at the same time both diversification of MHC class Ia molecules and homogenisation of the whole pool of MHC class I molecules (Ia and Ib) involved in antigen presentation.

**Reviewers:**

This article was reviewed by Stephan Beck, Lutz Walter and Pierre Pontarotti.

## Open peer review

Reviewed by Stephan Beck, Lutz Walter and Pierre Pontarotti.

For the full reviews, please go to the Reviewers' comments section.

## Background

A major histocompatibility complex (MHC) is found in the genome of all vertebrates from cartilaginous fish to mammals. This region, which covers over two megabases in the mammals where it has been studied [1], contains a large multigene family that encodes the membrane-bound glycoproteins known as MHC molecules. These molecules are not only crucial for establishing and controlling adaptive immune responses but also play important roles in many aspects of innate immunity. During evolution, a *bona fide *MHC region first appeared with the jawed vertebrates (gnathostomes) and correlates with the appearance of an adaptive immune system [2].

MHC molecules are frequently classified into three major groups. Firstly, class Ia molecules, also called classical class I molecules, are expressed at high levels on the surface of most nucleated cells. They present peptide antigens, derived mostly from intracellular proteins, to CD8+ cytotoxic T lymphocytes. This targets for destruction cells infected by intracellular pathogens, in particular viruses. The extraordinary allelic polymorphism of class Ia molecules at the level of their peptide binding region (PBR) is broadly perceived as a mechanism to counter the adaptive capacity of viruses.

Second, class Ib, or non-classical class I molecules, are usually expressed at lower levels than their classical counterparts, and show a more or less restricted tissue distribution. Within individual species, class Ib molecules exist in either a single or a few different allelic forms, and this relates to the fact that their role is to present ligands that are much less subject to genetic variation than peptides of viral origin [3]. Although this classification of MHC class I molecules into classical and non-classical is quite convenient, it should be noted that, for certain MHC class I molecules, the line between these two classes is difficult to draw.

The best characterised class Ib molecules present the leader peptides of class Ia molecules to natural killer (NK) cells, that recognise them with their CD94-NKG2A and CD94-NKG2B inhibitory or CD94-NKG2C activating receptors. This allows the immune system to counter the tendency of many intracellular pathogens to block the production of MHC class I molecules [3]. These class Ib molecules, which we will refer to as the CD94L family, are called HLA-E in human, H2-Qa1 in mouse, and RT-BM1 in rat. Such CD94L molecules have been identified in all rodent and primate species where MHC molecules have been extensively characterised, which hints at their important role(s).

The CD94L are, however, not the only MHC molecules regulating the lytic activity of NK cells: many other class Ia and class Ib molecules are also involved, through interactions with numerous activatory and inhibitory receptors on the surface of NK cells (see [4-6] for reviews).

The third group, class II molecules, are expressed mostly on professional antigen-presenting cells such as dendritic cells, macrophages and B cells. These molecules present peptides processed from proteins of extracellular origin to CD4+ regulatory T lymphocytes.

Although MHC molecules are encoded by the most rapidly evolving and polymorphic genes in vertebrate genomes, the MHC is, curiously, also a site of extraordinary conservation as regards the structure and functions of MHC molecules [7,8] as well as at the genomic level [1,2,9]. The evolution of CD94L molecules being much slower than their classical counterparts [10], this allows us to decipher patterns of evolution that are apparently paradoxical. Many other multigene families probably undergo processes similar to those we study here, but would be much less apparent than in the very diverse and actively evolving family of MHC class I molecules.

**Figure 1:**
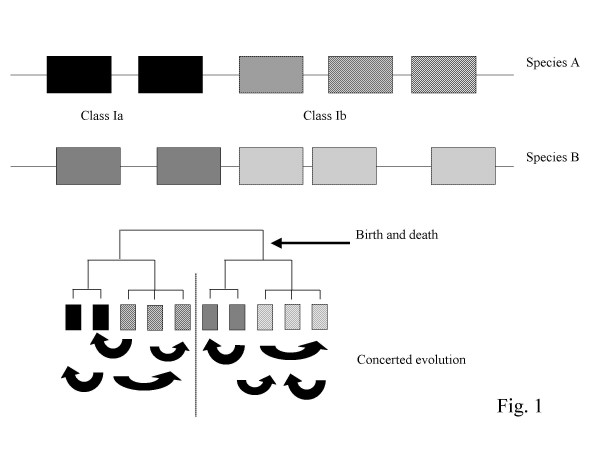
**Schematic representation of the 'birth an death' and the 'concerted evolution' models**. In phylogenetic analyses, class Ia (solid boxes) and class Ib (hatched) molecules within a species are more similar to each other than to class Ia or class Ib molecules in another species. According to the 'birth and death' model, this reflects the propensity of MHC molecules to derive from a single ancestral sequence by successive gene duplications. Another explanation, the 'concerted evolution' model, is that class Ia and class Ib molecules tend to become like each other due to frequent events of gene conversion, without necessarily deriving from a single sequence.

One of the most striking features of MHC class I molecules is that the sequences of classical (class Ia) and non-classical (class Ib) MHC class I molecules within a species are usually more closely related to one another than to their respective homologues in other species (Fig. 1) [11,12]. Two main hypotheses have been proposed to explain this apparent paradox: the 'concerted evolution' hypothesis and the 'evolution by birth and death' hypothesis.

*The concerted evolution hypothesi*s, on the one hand, suggests that frequent gene conversion events (i.e. non-homologous inter-locus recombination) homogenise the various sequences within multigene families. This means that the similarity among homologous genes is maintained over time, or will even tend to increase. Concerted evolution is a universal biological phenomenon. In species ranging from eukaryotes (including mammals) to prokaryotes, most multigene families thus far examined undergo concerted evolution [13]. Sequence homogenisation through extensive gene conversion was initially proposed by Baltimore to explain the homology observed between MHC genes within a species [14]. This hypothesis gained further support from Rada *et al*. when they compared sequences of rat and mouse MHC class I genes, and found evidence suggesting homogenisation of sequence motifs between distant loci on either side of the class II region [15]. More recently, work by Edwards and others has suggested that MHC molecules of birds could also undergo concerted evolution [16]. Concerted evolution of MHC molecules could explain why MHC molecules from separate loci within one species can be like one another without necessarily deriving from a common ancestral locus.

*The evolution by birth and death hypothesis*, on the other hand, proposes that strong homologies among MHC class I genes within a species can only arise when the entire class I gene pool re-evolves from a single sequence by successive gene duplications [11,12,17-19].

Historically, the two most likely, non mutually exclusive, explanations for the intra-species homology found within almost all multigene families have been gene amplification by repeated unequal crossing overs and gene conversion [20-22].

Genes for MHC molecules do indeed undergo very active expansion ('birth') and contraction ('death'), as testified by the extreme variability in the number of the various loci coding for different MHC molecules, even within species [18,23,24]. For example, depending on the strains, there are one or two functional class Ia loci in rats, two or three in mice, and three in humans.

The genes for class Ia molecules are also remarkably mobile within the MHC. For example, the human class Ia loci HLA-A, HLA-B and HLA-C occupy completely different positions in the MHC than those for RT1-A molecules, their functional homologues in rat. This gene 'jumping' is probably the result of regional duplications followed by loss of one of the original loci over the course of evolution. MHC genes therefore have an undeniable tendency to duplicate and disappear [25,26], consistent with the 'birth and death' idea.

One particular observation is, however, difficult to reconcile with evolutionary processes based solely on birth and death mechanisms as the sole explanation for the intra-species homologies of MHC molecules: although the CD94L molecules HLA-E and H2-Qa1 harbour certain structural similarities and clearly have the same function, HLA-E is, overall, more closely related to the human class Ia sequences (HLA-A, HLA-B and HLA-C), and H2-Qa1 to the mouse ones (H2-K, H2-D and H2-L). Given their views that, within species, the pool of MHC class I genes tends to derive by successive duplications of a single locus, Yeager, Kumar and Hughes concluded that HLA-E and H2-Qa1 must have arisen as a result of convergent evolution, *i.e*. that evolution produced molecules with similar functions twice independently [27].

While we were documenting the sequence variability of the rat CD94L class Ib molecule RT-BM1 among rat strains, we compared the rat class Ia and CD94L sequences to those of mouse and human [28]. In doing so, we noticed that the sequences of exon 4 (which codes for the membrane proximal α3 domain of the extracellular portion of class I molecules) were particularly similar between class Ia and class Ib sequences within species. On the other hand, comparing the sequences of exons 2 and 3 (coding for the PBR) of RT-BM1, H2-Qa1 and HLA-E suggested an orthologous relationship between these three genes, *i.e*. that all three molecules derived from a common ancestor. The logical consequence of this assertion was that the α3 domain homologies observed within each of the three species could only have arisen as a result of concerted evolution, *i.e*. by homogenisation of separate loci within species.

In this study, we have carried out a much more extensive comparison of the sequences of CD94L from four primate and four rodent species with those of class Ia sequences from the same species. The results of these comparisons allow us to conclude that separate loci for MHC molecules do undeniably undergo concerted evolution within species, with different portions of the molecules evolving differently. Since the 'concerted evolution' and the 'birth and death' hypotheses are not mutually exclusive, our results are in agreement with a model where both phenomena contribute to the homology of MHC molecules observed within species. This leads us to discuss concepts of why the MHC exists, and how it evolved and will keep evolving.

## Results

### HLA-E, H2-Qa1 and RT-BM1 occupy similar positions within the MHC

Early comparative maps of the human and mouse MHC placed the HLA-E locus in a much more telomeric position than the H2-Qa1 locus [for example, see Figure 3 of [9]]. This contributed to the general perception that these two loci were unlikely to occupy conserved syntenic positions, and were therefore unlikely to be orthologues (we use this term to indicate that sequences derived from the same ancestral locus). If the positions of invariant genes within the MHC region are taken as a reference point [29], however, the CD94L loci HLA-E, H2-Qa1 (also called H2-T23) and RT-BM1 are all found in the very same genetic location: the portion of the MHC class I 'island' between *GNL1 *and *RPP21 *[30,31]. On the grounds of genetic mapping, therefore, there is no reason to believe that the primate and rodent loci for CD94L could not be *bona fide *orthologues.

**Figure 3:**
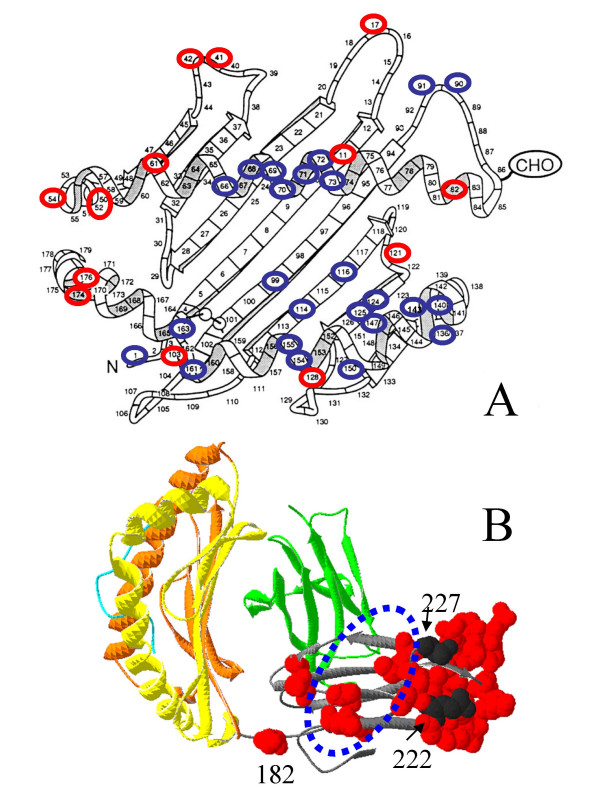
**Topologic distribution of the CD94L-specific and species-specific residues**. A) The locations of CD94L-specific (blue) and species-specific (red) residues on the schematised structure of an α1/α2 co-domain. B) The residues of the α3 domain that have undergone intra-species homogenisation in at least one of the eight species studied (red numbers in Fig. 2) are represented in red 'spacefill' mode. Residues 222 and 227, which influence interactions of class I molecules respectively with tapasin and calreticulin, appear in black (and not red). The structure used is that of the rat MHC molecule RT1-A^a^, bound to a 13-mer peptide of mitochondrial origin (acc. 1ED3) [82]. Representation of the 3D structure was generated with the Deep View Swiss-PdbViewer. The α1 domain is in orange, α2 in yellow, α3 in grey, beta 2 microglobulin in green and the peptide in light blue. The supine orientation of the molecule used here corresponds to that which is naturally adopted by MHC molecules on the plasma membrane, according to Mitra et al. [34]. The area indicated by blue dots corresponds to the footprint of the CD8 molecule [33].

### Certain residues are CD94L-specific, and others are homogenised within species

To investigate the evolutionary relationship between the CD94L and class Ia loci, we searched the nucleotide database to identify species in which sequences were available covering the whole extracellular portions (domains α1, α2 and α3) of both CD94L and class Ia molecules. This search yielded four rodent species [mouse (*Mus musculus*), rat (*Rattus norvegicus*), Chinese hamster (*Cricetulus griseus*), deer mouse (*Peromyscus maniculatis*)] and four primate species [human *(Homo sapiens*), chimpanzee (*Pan troglodytes*), Rhesus macaque (*Macaca mulatta*), and cotton-top tamarin (*Saguinus oedipus*)]. CD94L and/or class Ia sequences are available for many other rodent and primate species, but either as partial sequences, or only for either a CD94L or class Ia molecule(s). Attempts to identify potential CD94L molecules in species outside of the primate and rodent genera by homology searches in sequence databases yielded no promising candidates (not shown).

For each of the species listed above, in addition to the CD94L sequence, we picked one representative of each of the characterised class Ia loci. For the hamster and deer mouse, because the definite locus information was lacking, we picked sequences that were as different from one another as possible (two for hamster, and three for deer mouse). To extend our investigation beyond the CD94L-type class Ib molecules, we also included the rat RT1-M3 and mouse H2-M3 class Ib sequences. RT1-M3 and H2-M3 are clear orthologues, and are both specialised in the presentation of bacterial N-formylated peptides [32].

**Figure 2:**
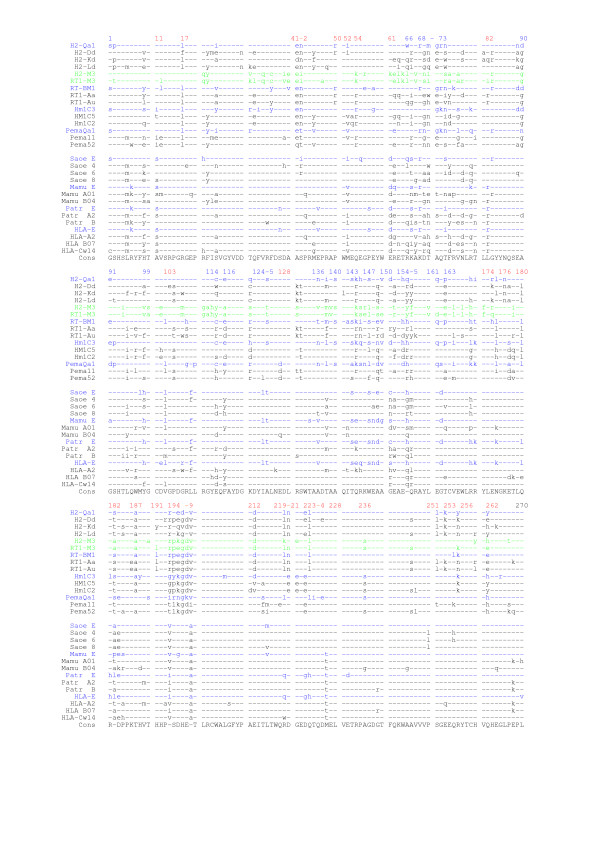
**Identification of species-specific and CD94L-specific residues**. Comparison of the CD94L protein sequences (blue) with those of the class Ia molecules of their respective species (black). The sequences of the murine class Ib molecules M3, which are orthologous between mouse and rat were also included (green). Sequences from rodent are on the top 15 lines, and from primates on the lower 14 the alignment (see Materials and Methods for key to the abbreviations used). The 'pretty' output of the GCG software was used to generate this alignment of the MHC molecules extra-cellular portions (α1 domain: 1–90, α2: 91–180, α3: 181–270). Positions in agreement with the consensus are indicated by -. The positions of CD94L-specific residues, *i.e*. those that were found in at least 3 of the 4 CD94L rodent or primate sequences and not in the corresponding class Ia sequences are indicated by a blue number above the alignment. Conversely, certain residues were identified that differed from the general consensus, but were common to all the sequences of at least one species. These positions, where intra-species homogenisation has taken place, are indicated by a red number above the alignment.

Altogether, we selected 29 sequences, and aligned the protein sequences of their extracellular domains (Fig. 2). All these sequences are closely homologous to one another, with no gaps or deletions in the alignment, and there is perfect consensus between all the class I molecules at 87 positions (32.2%).

At 24 positions along the sequence (indicated by a blue number above the alignment), some residues have clearly been conserved among the CD94L molecules (in blue letters) within either the rodent or the primate orders (for example, see positions 116 and 140 for rodent CD94L, and 124 and125 for primate CD94L). At six of these positions (68, 69, 70, 143, 147, 155), the same residues are found in either seven or all eight of the primate and rodent CD94L sequences.

Given the previously reported observation that HLA-E, H2-Qa1 and RT-BM1 are each more closely related to their respective class Ia counterparts, we also inspected the alignment to get an idea of the localisation of the species-specific residues (Because the number of class Ia sequences included in this alignment is very small compared to all those known in the different species, the list of residues we identified in this manner should, however, only be considered as indicative rather than exhaustive and/or definitive). Such positions, where all the shown class Ia and Ib sequences from at least one species are identical to one another but diverge from the general consensus, are indicated by a red number above the alignment (for example, position 52 is a valine in all three macaque sequences, and position 54 is an arginine in all three hamster sequences). Fourteen such positions occur within the first 180 residues, which corresponds to the α1/α2 codomain that constitutes the PBR. There is very little room for doubt that all four primate CD94L genes descend from a common ancestral gene, and similarly for all four rodent CD94L genes. Both in the primate and in the rodent ancestral species, there must, therefore, already have been a co-existence of the loci for ancestral CD94L molecules and for ancestral class Ia molecules. The most likely explanation for residues found in the class Ia and CD94L molecules of only one species is the occurrence of gene conversion between the various MHC class I genes in that species, including those for class Ia and CD94L molecules, resulting in localised homogenisations, which would ultimately lead to concerted evolution.

Remarkably, within the α3 domain, which separates the PBR from the transmembrane domain (last 90 residues of the alignment), 22 positions have evolved in a species-related manner, and no single position is found among either the rodent or primate CD94L groups despite the fact that separate loci for class Ia and CD94L must have pre-existed the species' radiation within each genus.

From these observations, we conclude that, at least for the α3 domain, the CD94L have all undergone intra-species concerted evolution with their respective class Ia molecules. This phenomenon is very evident among rodent sequences, but it can also be observed within the primate ones (e.g. at positions 182, 194 and 228). The identification of 14 positions along the α1/α2 codomain that have undergone similar intra-species homogenisation further suggests that this type of phenomenon is likely to be true also of sequences outside exon 4.

### Different topological distributions of the CD94L- and species-specific residues

We analysed the locations on the MHC class I molecule of those residues that have been conserved among CD94L molecules and those that evolve in a species related manner (Fig. 3A and 3B). The residues that appear to undergo intra-species concerted evolution are all located outside the PBR, whereas most of the residues that are conserved amongst CD94L molecules are situated at positions within the PBR. Three noticeable exceptions are positions 1, 90 and 91.

The observation that the residues of the α1/α2 codomain that undergo concerted evolution mostly tend to occupy positions on peripheral loops suggests that these positions may correspond to sites of interaction of the class I molecules with other proteins.

Remarkably, most of the α3 domain residues that have undergone concerted evolution occur furthest away from the PBR (Fig. 3B), and a handful of others lie at the CD8 interaction site (area indicated by a blue dotted oval) [33]. According to the recent report by Mitra et al[34], MHC class I molecules are not 'standing up' on the plasma membrane as is generally presumed, but adopt a supine position, as shown here. In this conformation, the portion of the α3 domain where residues have a strong tendency to undergo concerted evolution would be eminently accessible to interactions with one or several molecular partners.

### Different portions of MHC class I molecules undergo different patterns of evolution

**Figure 4:**
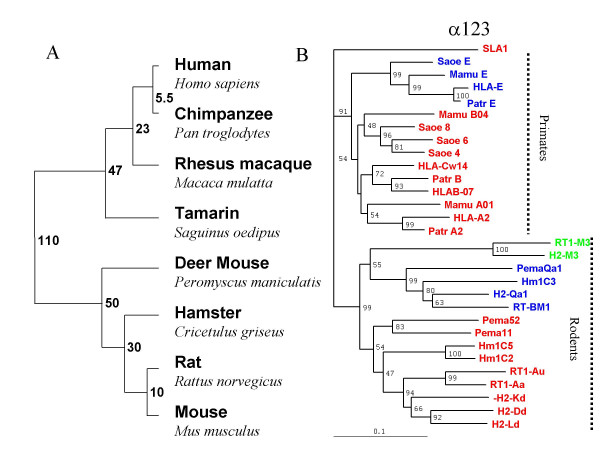
**When whole length sequences are compared, class Ib molecules cluster by function within taxa**. A) A schematic representation of the divergence of the eight species studied here. The divergence times, indicated in millions of years, are those estimated from the fossil record. B) A comparison of the whole length of the aligned protein sequences shown in Fig. 2 (270 aa). Percentage bootstrapping support values are indicated of the branches (values above 60% are generally considered as highly significant). Class Ia molecules appear in red. SLA is a pig class Ia molecule chosen as an outlier, since the split with the ancestor of pigs occurred much earlier than that between primates and rodents [83]. CD94L molecules are shown in blue, and M3 molecules in green.

Using the protein alignment in Figure 2, we carried out successive phylogenetic comparisons focusing on various portions of the proteins. The tree shown in Figure 4A is a graphical representation of the widely accepted divergence pattern of the eight species in this study, together with the divergence times estimated according to the fossil record. We first compared the entire extracellular portions (270 amino acids; α1+α2+α3 domains) of the 29 sequences together with the pig class Ia sequence SLAI (used as an 'outlier') (Fig. 4B). Our findings confirm the previously reported observation that class Ia molecules (red) and class Ib molecules (blue and green) co-segregate within an order [11,12].

Among the class Ia molecules the only orthologous relationships suggested by this analysis are between the human and chimpanzee B loci (HLA-B07 and Patr B), and human, chimpanzee and macaque A loci (HLA-A2, Patr A2 and Mamu A01), with no locus orthology suggested within rodents. Among the class Ib molecules, however, the independent groupings of CD94L sequences for primates and for rodents establishes beyond reasonable doubt that, within each group, all four loci must derive from a common ancestral CD94L locus. The fact that the comparison of primate sequences strongly suggests that the four CD94L are orthologues, whereas this is much less clear for the corresponding class Ia sequences confirms previous reports that primate CD94L molecules are much more evolutionarily conserved than are class Ia molecules [10,35,36], and extends this observation of high conservation to rodent CD94L and to the M3 class Ib molecules found in mouse and rat. If HLA-E and H2-Qa1 arose by convergent evolution, this must not only have taken place earlier than the intra-order species divergence, roughly 50 million years ago, and after the primate-rodent split 100 million years ago.

**Figure 5:**
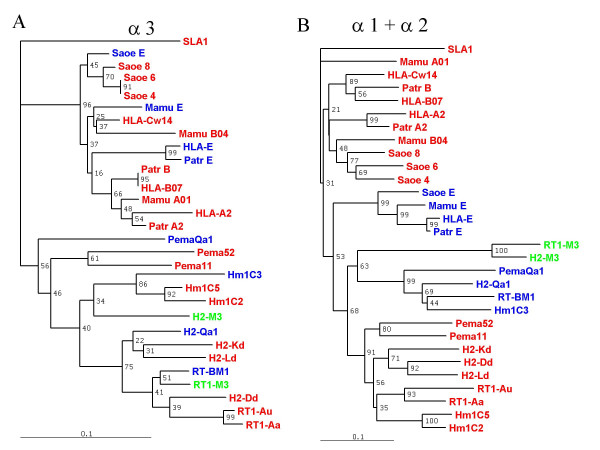
**Intra-species concerted evolution is particularly prominent in the α3 domain**. A) The tree was generated using the protein sequences of the α3 domain (last 90 aa of the aligned protein sequences shown in Fig. 2). B) The tree was generated using the protein sequences for the α1/α2 co-domain (first 180 aa of the aligned protein sequences shown in Fig. 2). Percentage bootstrapping support values above 60% are generally considered as highly significant. Class Ia molecules appear in red. SLA is a pig class Ia molecule chosen as an outlier, since the split with the ancestor of pigs occurred much earlier than that between primates and rodents [83]. CD94L molecules are shown in blue, and M3 molecules in green.

Given the observations collected from the sequence alignment in Figure 2, which suggested that the sequence of the α3 domain may evolve differently from that of the α1/α2 codomain, we next generated phylogenetic trees to compare separately the evolution of these domains (Fig. 5). Comparison of the α3 domains reveals a strong tendency of the class Ia sequences (red) and class Ib (blue and green) to group by species. This observation confirms our earlier conclusion from direct inspection of the sequence alignment;*i.e*. that concerted evolution within species does clearly take place between class Ia and class Ib sequences, at least for the sequences of the α3 domains. This is supported with very strong significance by the groupings of the tamarin and hamster CD94L molecules with their respective class Ia counterparts rather than with their respective primate and rodent CD94L functional homologues. The clade containing all the murine MHC molecules, class Ia, M3 and CD94L also provides a further clear proof of the intra-species homogenisation of the α3 domains, although, in this latter case, the H2-D^d ^molecule clusters with the rat MHC class Ia sequences rather than with those of other mouse class Ia.

We then constructed a phylogenetic tree for the first 180 amino acids that constitute the α1–α2 codomain (Fig 5B). The most remarkable difference between the tree shown in Figure 4B and this one is that the removal of the species-specific sequences of the α3 domain results in the rodent and primate CD94L molecules (blue) falling much closer together, and away from the clades of their respective class Ia counterparts. This finding already suggests that these two sets of class Ib molecules may well share a common ancestor.

The presence of the rat and mouse M3 molecules (green) on the same branch as the group of rodent CD94L could indicate that the M3 and CD94L loci arose by gene duplication in a rodent ancestor before the rat-mouse divergence. An alternative explanation is that the CD94L and M3 loci have coexisted long enough for concerted evolution to drive their homogenisation. Yet another interpretation – turning the tree on its head – is that the M3 molecules may not only go back a long time, but the structural requirements imposed by their function may be sufficiently tight that their evolution could have been much slower than that of the other molecules on the tree. In this case, these sequences would be closer to an 'ancestral' MHC sequence, with the CD94L sequences moving slowly away from it, and those of class Ia molecules much faster. The clustering of the mouse H2-M3 α3 domain sequence with those of hamster sequences rather than that of other mouse sequences could be seen as a further, albeit weak, argument in support of this argument.

Given the observation that certain residues of the α1–α2 codomain that are situated outside the PBR tend to evolve by species, we carried out separate comparisons for those 67 residues that are generally considered as being involved in the PBR, and for the 113 residues outside the antigen recognition site (ROARS; see Materials and Methods for the precise list).

**Figure 6:**
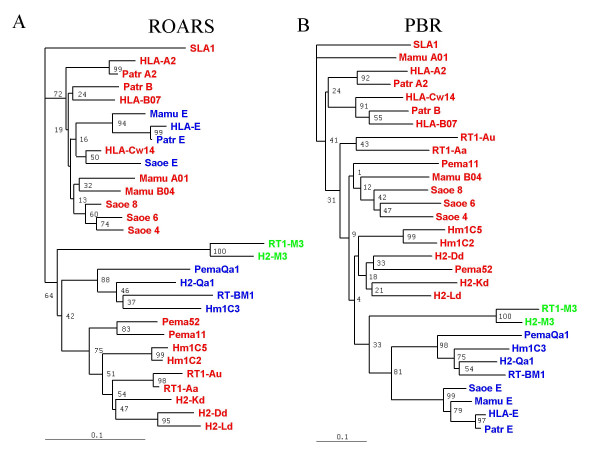
**In the α1/α2 co-domain, residues inside and outside the PBR evolve differently**. A) The tree was generated by comparing the 113 residues of the α1/α2 co-domain that fall outside of the antigen recognition site (ROARS). B) The tree was generated by comparing the 67 residues that take part in the PBR. Percentage bootstrapping support values above 60% are generally considered as highly significant. Class Ia molecules appear in red. SLA is a pig class Ia molecule chosen as an outlier, since the split with the ancestor of pigs occurred much earlier than that between primates and rodents [83]. CD94L molecules are shown in blue, and M3 molecules in green.

The tree obtained by comparing the ROARS (Fig. 6A) has roughly the same overall structure as that derived from comparing the three domains together (Fig. 4B), with the rodent sequences clearly separated from the primates. The class Ia molecules (red) have a strong tendency to cluster by species, and the class Ib (blue) by function within each order, the exception being Saoe E that clusters with HLA-C. This clustering of class Ia loci by species is probably in good part explained by gene duplication and contraction, as proposed in the birth and death model [18]. Given our observation of certain species-specific residues harboured by both class Ia and CD94L molecules, however, we believe that concerted evolution within species could occur significantly for these ROARS, thereby also contributing to the sequences' tendency to group by species.

Gene conversion can result in the transfer of as few as half a dozen nucleotides to as many as several hundred [37]. For the sequences coding the α1–α2 codomain, the interspersed arrangement of PBR and ROARS residues must greatly influence the size of the DNA segments that can be exchanged to result in ROARS homogenisation. For class Ib molecules, the need to preserve their defined antigen-binding specificity would mean that PBR residues could not undergo gene conversion with other loci. The size of the DNA segments being exchanged would then be limited to those fitting in between PBR residues, resulting in a slower rate of evolution than for the α3 domain, where the size restriction would not apply. For class Ia molecules, however, the very intense evolutionary processes that drives their PBR residues towards heterogeneity would result in an acceleration of the homogenisation of ROARS compared to the α3 domain residues.

Comparison of this tree (Fig. 6A) to that for α3 domains (Fig. 5A) indeed hints that class Ia ROARS sequences homogenise even faster than their α3 domains. Firstly, mouse H2-D^d ^falls among rat sequences in the α3 domain analysis but clusters with the other mouse class Ia molecules for the ROARS. Similarly, the Mamu A01 sequence clusters with human and chimpanzee A sequences for the α3 domain but with the Mamu B04 sequence for the ROARS (albeit with low support values).

When the 67 PBR residues are compared (Fig. 6B), all CD94L (blue) fall in one clearly defined group. This grouping could be explained either by convergent evolution, or by the fact that all eight CD94L genes derive from a common ancestor. Since the murine and human CD94L loci occupy conserved syntenic positions within the MHC, and since concerted evolution can explain the homologies of MHC class I sequences found overall within species, we conclude that all eight CD94L molecules almost certainly derive from a common ancestor, without the need to call upon convergent evolution. A further argument supporting this conclusion is the arrangement of the CD94L sequences on this tree that matches exactly the divergence of the species (Fig. 4A). Comparison of the PBR of the class Ia molecules (red) gives an indication of their exuberant evolution, with some primate sequences (Saoe 4, Saoe 6, Saoe 8 and Mamu B04) intermingled with rodent sequences (with, however, exceedingly low support values for the branches), and Mamu-A01 resembling most closely the pig class Ia molecule we picked as an outlier. From this type of observation, we conclude that phylogenetic trees are not necessarily the best tool to decipher the evolution of residues that undergo extremely rapid evolution such as the PBR residues of class Ia molecules.

### The PBR residues of primate and rodent CD94L molecules have been actively conserved from a common ancestral sequence

Comparing the rates of synonymous versus non-synonymous (ds/dn) substitutions in nucleic acid sequences has been used to great effect to investigate the nature of the selective pressures that drive the evolution of MHC molecules, particularly to demonstrate that the PBR residues of class Ia molecules are under positive selective pressure [17]. If our conclusion that the rodent and primate CD94L molecules derived from a common ancestor is correct, and the antigen presenting function of these molecule has been conserved for over 50 million years, then we predict that their PBR residues would show signs of negative selective pressure.

To test this prediction, we carried out comparisons of synonymous versus non-synonymous substitutions for the codons corresponding to the PBR residues. With this type of analysis, sequences under negative selective pressure are expected to have ds/dn values greater than 1, and those under positive pressure less than 1. As can be seen in Table 1, a comparison between the corresponding 19 class Ia sequences gave an average ds/dn value of 0.87, confirming that PBR residues of class Ia molecules evolve under positive selective pressure [17]. Conversely, the global average of the ds/dn values found for the eight CD94L molecules was 3.02, suggesting negative selective pressure, or, in other words, that PBR residues have been actively conserved in CD94L molecules since deriving from a common ancestor. More importantly, we found that the averages for inter-order and intra-order CD94L comparisons were roughly comparable (3.40 and 2.78, respectively), with an interspersed distribution of individual intra- and inter-order ds/dn values.

**Table 1:** A table showing ds/dn comparison for PBR residues of CD94L. Comparing synonymous versus non-synonymous substitutions for PBR residues confirms that rodent and primate CD94L sequences all derive from a common ancestor.

		**ds/dn values**	*Rank among 406 pairwise comparisons*
Saoe E	Patr E	**6,35**	*1*
RT-BM1	Hm1C3	**5,29**	*2*
Saoe E	HLA-E	**4,94**	*3*
H2-Qa1	RT-BM1	**4,59**	*5*
Saoe E	MaMu E	**4,13**	*6*
Hm1C3	HLA-E	3,7	*9*
RT-BM1	Saoe E	3,65	*10*
Hm1C3	MaMu E	3,37	*13*
Hm1C3	Patr E	3,25	*15*
H2-Qa1	Saoe E	3,24	*16*
H2-Qa1	HLA-E	2,99	*21*
RT-BM1	Patr E	2,95	*23*
H2-Qa1	MaMu E	2,89	*25*
RT-BM1	HLA-E	2,88	*27*
RT-BM1	PemaQa1	**2,83**	*31*
H2-Qa1	Hm1C3	**2,8**	*33*
H2-Qa1	Patr E	2,79	*36*
PemaQa1	Patr E	2,59	*47*
RT-BM1	MaMu E	2,49	*55*
PemaQa1	HLA-E	2,47	*58*
PemaQa1	MaMu E	2,4	*61*
Hm1C3	Saoe E	2,27	*69*
MaMu E	HLA-E	**2,21**	*77*
Patr E	HLA-E	1,9	*100*
MaMu E	Patr E	**1,62**	*134*
PemaQa1	Saoe E	1,51	*151*
H2-Qa1	PemaQa1	**1,4**	*165*
Hm1C3	PemaQa1	**1,19**	*217*
**averages**			
for all 29 sequences	406 comparisons	1,42	
for 19 class Ia sequences	171 comparisons	0,87	
for 8 CD94L sequences	global(28 comparisons)	3,02	
	11 intra-genus	3,40	
	17 inter-genus	2,78	

ds/dn ratios were calculated for the PBR residues of aligned nucleic sequences corresponding to all 29 proteins appearing in Fig. 2, yielding 406 individual pairwise comparisons (see MαM). For the sake of space and clarity, only the ds/dn values obtained for comparisons among CD94L molecules are shown here, as well as their rank within those 406 comparisons. Values for intra-taxa comparisons are shown in bold (i.e. primate vs primate or rodent vs rodent CD94L).

Although the statistical interpretation of this distribution is difficult, it is certainly consistent with our conclusion that all eight CD94L most likely derive from a common CD94L ancestor molecule, and have all evolved under similar global evolutionary pressures since the rodent-primate radiation that took place over 100 million years ago.

## Discussion

### Concerted evolution to reinterpret previous data

Since the polymorphism of the HLA-E locus apparently predates most HLA-A and HLA-B polymorphism [10], HLA-E can be considered as quite an ancient locus, which fits with our conclusion that it must have been present in the rodent/primate ancestor. In 1989, Hughes and Nei pointed to a probable interlocus exchange of the whole exon 4, coding for the α3 domain, between HLA-A and HLA-E [38]. The sequence alignment shown in Figure 1 of their paper (see Additional file 1), however, is more consistent with gene conversion than with an overall exon swap by recombination between HLA-A and HLA-E. Over the first 21 nucleotides of exon 4 (red box), HLA-E is much more closely related to HLA-B, HLA-C and the Patr B sequence than to HLA-A or Patr A. Over the following 93 nucleotides (blue box), all six sequences are almost completely homologous to one another. The relatedness of the HLA-E α3 domain to those of HLA-A and Patr A is, in fact, due almost entirely to eight sites in the subsequent 99 nucleotides where the HLA-B, HLA-C and Patr B sequences diverge from these three sequences. The following 12 nucleotides are completely homologous between the six sequences (blue box), and over the last 48 nucleotides of exon 4 (red box), the HLA-E sequence matches once again better with HLA-C than with HLA-A. Given that the length of the DNA segments exchanged by gene conversion can concern any number from less than 10 to several hundred nucleotides [37], this 'mosaicism' of the human and chimpanzee sequences is, therefore, much more suggestive of events of gene conversion than of exon swapping, and the extensive occurrence of such events appears to be the most likely source of the concerted evolution we witness for the α3 domain.

The fact that gene conversion contributes to the diversity of MHC molecules is broadly accepted. The frequency of past gene conversion events in human and chimpanzee MHC class I sequences was even evaluated statistically by Kuhner et al. [39], and matching of the data on polymorphism obtained in mammals to mathematical simulations has suggested that the rate of allelic conversion is relatively high [40]. Although gene conversion can contribute to the generation of new MHC class I alleles by shuffling DNA sequence motifs, thereby enhancing heterogeneity, the very same mechanism can also result in homogenisation of sequences within a multigene family. Since gene conversion does not actually mutate DNA but simply shuffles motifs between existing loci, we can see that this phenomenon has no intrinsic effect on increasing or decreasing heterogeneity of individual residues. What gene conversion actually does is to accelerate the rate of evolution of MHC molecules, and it is the selective pressures on the function of MHC molecules that must drive certain portions of these molecules towards homogeneity or heterogeneity.

### Concerted evolution and protein function

Several hypotheses have been proposed to explain the fact that MHC molecules are the most diverse and fastest evolving genes in mammalian genomes, including pathogen driven evolution, high spontaneous mutation rate, frequent gene conversion, mating preferences and materno-foetal incompatibility [41]. These hypotheses are not mutually exclusive and probably all contribute to a certain extent to MHC diversity, but the main driving force is clearly the stringent selective pressure imposed by infectious pathogens. Several models have been put forward to explain how the pressure of fast evolving pathogens drives MHC diversity, for example balancing selection, over dominant selection and rare allele advantage All these models are reviewed and discussed in a recent review by Jeffery and Bagham [42].

In their turn, many pathogens have devised several clever ways to block antigen presentation by making inhibitors that bind either directly to MHC molecules outside the PBR or to other molecular partners involved in antigen processing, presentation and recognition [43,44], providing another level of selective pressure by pathogens on the immune system. Many of these proteins [e.g. the transporter for antigen processing (TAP), tapasin, calnexin, calreticulin, beta 2 microglobulin (β2M), CD8 and the T cell receptor for antigen (TCR)] must, therefore, also be under considerable evolutionary pressure to evade these inhibitory mechanisms.

If a pathogen evolves to block the antigen presentation/recognition pathway, individuals carrying mutations that allow them to evade this blockage will have a sizeable advantage to fight off the infection. Such advantageous mutations might occur either in one of the multiple loci coding for MHC class I molecules, or in one of the genes encoding proteins involved in the antigen presentation/recognition pathway (PRIAP). If a mutated form of a PRIAP arises as a result of pathogen selection, one potential consequence would be that the pool of MHC class I molecules in that species would no longer be optimally suited to interact with that PRIAP mutant. Evolutionary pressures would, in turn, favour individuals harbouring MHC molecules adapted to interact better with these new PRIAP forms. Such co-evolution has been reported for the genetically linked TAP and MHC class Ia loci in the rat [45] and the chicken [46]. In these circumstances gene conversion could spread advantageous motifs between MHC molecules within a given species, ultimately resulting in their concerted evolution.

In strong support of this model, in our study, all the positions evocative of species-specific evolution correspond to residues that would be readily accessible to interactions with other proteins, be they PRIAPs or inhibitors of antigen presentation derived from infectious pathogens (or both). Regarding the remarkable congregation of species-specific residues in the portion of the α3 domain furthest away from the PBR, this portion of MHC class I molecules is known to be involved with interactions with the peptide-loading machinery; position 222 is involved in interactions with tapasin [47] and position 227 with calreticulin [48]. Residue 182, which also undergoes intra-species homogenisation, is located next to the site bound by the inhibitory protein US2 from human cytomegalovirus [49]. Our observation that three positions in the α1–α2 codomain that clearly lie outside of the PBR (1, 90 and 91) tend to be conserved among CD94L molecules could indicate sites of interactions with partners specific to those molecules, possibly with the CD94-NKG2 receptors themselves.

NK receptors are amazingly diverse both within and between species, and they have almost certainly co-evolved with the MHC molecules they recognise [50-53]. Certain residues of MHC class I molecules that evolve in a species-specific manner may therefore be involved in interactions with NK receptors.

Our results suggest that, in MHC molecules, different subsets of residues are under different selective pressures. As previously shown by Hughes and Nei, the PBR residues of class Ia molecules, which present highly variable antigens, are under positive selective pressure, favouring heterogeneity of their antigen-binding properties [17]. Conversely, in class Ib molecules, which present precisely defined antigens such as N-formylated peptides or the leader peptides of class Ia molecules, the same PBR residues are under negative selective pressure to ensure maintenance of function. Similarly, for class Ia molecules, the ROARS residues of the α1 and α2 domains undergo frequent gene conversion and evolve fast, whereas in class Ib molecules, which are less variable, the ROARS residues evolve more slowly. We predict that the residues of the α3 domain will evolve at similar rates in class Ia and class Ib molecules, between those of the fast class Ia and the slow class Ib ROARS.

By contributing to a better understanding of how MHC molecules evolve, we hope that our results may help to decipher where and how our adaptive immune system arose, and keeps evolving in the face of the permanent challenge of infectious organisms or, as the case may be, the lack of them that may well favour allergic or auto-immune conditions.

## Materials and methods

Protein and DNA sequences of MHC class I molecules were all obtained from the database with the accession numbers provided in table 2. Over the region studied (α1 to α3 domains encoded by exons 2, 3 and 4), we made sure that all the sequences chosen had exactly the same length, with no insertion of gaps necessary, since introducing gaps can affect the value of the comparisons obtained [72]. We chose to compare protein rather than nucleic acid sequences for three main reasons: i) this circumvents the concern about potential differences in G/C contents at synonymous sites between class Ia and Ib sequences [11,73], ii) The sequences are shorter, and hence easier to compute, and the validity of the alignment is much easier to ascertain iii) The comparisons incorporate degrees of similarity between residues, which seems important when dealing with sequences under strong selective pressures.

**Table 2:** Accession numbers and bibliographic references of the sequences used for this work

Species	Class I sequences	Protein acc n°	DNA acc n°	Ref
Human	HLA-A2	386875	K02883.1	[54]
*Homo sapiens*	HLA-B7	1213467	U29057.1	
	HLA-Cw1403	1389552	D31817.1	[55]
	HLA-E	306852	M20022.1	[56]
Chimpanzee	Patr A2	122146	M30678.1	[57]
*Pan Troglodytes*	Patr B	38209	X13116.1	[58]
	Patr E	15011269	AF338354.1	[59]
Rhesus Macaque	Mamu A01	1255176	U50836.1	[60]
*Macaca mulatta*	Mamu B04	1399313	U41826.1	[61]
	Mamu E	1399333	U41837.1	[61]
Cotton-top tamarin	Saoe 4	226977	M63946.1	[62]
*Saguinus Oedipus*	Saoe 6	226973	M63950.1	[63]
	Saoe 8	226969	M63947.1	[62]
	Saoe E	3015548	AF004918.1	[36]
House mouse	H2-D^d^	AAA39581	L29190	[64]
*Mus musculus*	H2-K^d^	AAA39652	J00402.1	[65]
	H2-L^d^	199557	M33151.1	[66]
	H2-Qa1^d^	AAD31381.1	AF057279.1	[67]
	H2-M3	619937	U18797.1	[68]
Laboratory rat	RT1-A^u^	2887302	X82106.1	[69]
*Rattus Norvegicus*	RT1A^a^	1877416	M31018.1	[15]
	RT-BM1^c^	5640125	AJ243975.1	[28]
	RT1-M3	12621072	NM_022921.1	[32]
Chinese hamster	Hm1C5	21903710	AY064390.1	[70]
*Cricetulus Griseus*	Hm1C2	21903704	AY064387.1	[70]
	Hm1C3	21903706	AY064388.1	[70]
Deer mouse	Pema 52	531452	U12886.1	[71]
*Peromyscus maniculatis*	Pema 11	576636	U16846.1	[71]
	Pema Qa1	AAB17692.2	U12822.3	[71]
Pig *Sus scrofa*	SLA1	38503456	AY459306.1	

To assemble the list of residues taking part in the PBR, we were careful not to be influenced by the observations collected from the alignment shown in figure 2. Rather, we constituted a pooled list of all the residues that we could find in the literature as allegedly taking part in the PBR [27,74-76]. The resulting list of 67 residues is as follows: 5, 7, 9, 22–26, 34, 45, 57–59, 61–77, 80–82, 84, 95, 97, 99, 114, 116, 118, 123–124, 133, 143, 145–147 149–152, 154–163 165–167 169–171. The 113 ROARS residues are those between 1 and 180 that are not in the above list.

To generate the subset of residues for the various domains and sub-domains, we made use of the Seaview software [77], which is available for download from . Neighbour Joining (NJ) trees were then drawn with the phylip option of the clustalW software which can be downloaded together with Seaview. Support values for branches were obtained by 10000 boostrapping steps. For the sake of completeness of information, we chose to provide all the values obtained. Values above 60% are usually considered significant. Trees based on the same alignments were also generated with the puzzle 5.2 software [78], and gave very similar results to those shown in this paper (data not shown). Many other trees were also generated that incorporated many more MHC class I sequences than the ones that were picked pretty much at random for the work presented here. All these trees always gave the same overall indications as the ones shown here. We chose to present trees based on this restricted number of sequences for the sake of simplicity of presentation.

All trees were visualised with Treeview.

, [79]

Comparisons of synonymous vs non synonymous substitutions were carried out with the SNAP software [80], which is based on the method described by Nei and Gojobori [81], and is available online on the HIV sequence database NIH web server .

Representation of the RT1-A^a ^3D structure (acc 1ED3) was generated with the Deep View Swiss-PdbViewer 

## Authors' contributions

Virginie Rouillon carried out some of the initial comparison experiments, and identified, downloaded and installed all the various software programs used for this work.

Etienne Joly did everything else.

## Reviewers' comments

### Reviewer's report 1

#### Stephan Beck

In their manuscript Joly and Rouillon report new evidence for the hypothesis that MHC class I genes undergo concerted evolution through gene conversion (e.g. non-homologous recombination). In support, they analysed 8 classical class I genes (termed class Ia) and 8 non-classical class I genes (termed class Ib) from primates and rodents with focus on three class Ib genes to which they refer to as CD94L family (human HLA-E, mouse H2-Qa1 and rat RT-BM1). The results are comprehensively discussed within the context of various relevant hypotheses which adds a review-like flavour and greatly enhances the appeal of the manuscript.

Although some conclusions (and assumptions) are better supported than others, I only take issue with one particular point. Based on evidence I do not agree with, the authors assume the above mentioned CD94L family genes to represent orthologues and their many respective paralogues are not considered in subsequent analyses which may have affected some of the conclusions.

**Author response: ***Following this comment, and a suggestion made by Pierre Pontarotti on the phone, I have now modified the manuscript to remove the statement about 'the clear orthologous relationship of CD94L molecules within the primate or the rodent orders'. This is now replaced by 'There is very little room for doubt that all four primate CD94L genes descend from a common ancestral gene, and similarly for all four rodent CD94L genes'*.

On page 12, for instance, the authors conclude that at least the alpha 3 domains of the CD94L genes have all undergone intra-species concerted evolution with their respective class Ia molecules but not one of the 22 informative positions is shared across species as one would expect for orthologues. The logic conclusion would have to be that gene conversion did not occur in the ancestral (e.g. pre 80 mya) CD94L genes studied here. This is unlikely, as gene conversion has been demonstrated to be a general mechanism clearly predating the species studied here.

**Author response: ***Thanks to the process of 'open refereeing', I have been able to discuss this point with Stephan over the phone directly. His comment sprouted from some slight misunderstanding, which has now been lifted*.

The additional section (appended to main manuscript) does not really constitute a separate manuscript but adds further interesting points to the discussion and the key points could be summarized and included in the main manuscript.

**Author response: ***The solution to this has been to remove a sizeable portion of the discussion and to provide it as a clearly separate manuscript*.

*The paper itself now focuses on the demonstration that HLA-E and Qa1 are orthologues. It is now much shorter, easier to read, and the message is, I hope, much clearer*.

*The accompanying paper is now clearly labelled as 'hypothesis', and I have used it to regroup 4 topics of discussion touching on different aspects of MHC evolution that derive from the results obtained in the paper itself, but are not directly related to these results*.

### Reviewer's report 2

#### Lutz Walter

In this paper, Joly and Rouillon compare major histocompatibility complex (MHC) class I genes derived from human, non-human primates, and rodents. Based on multiple sequence alignments and phylogenetic tree reconstructions, the authors conclude that the MHC class I genes in these species are subject to concerted evolution by means of gene conversion.

One main point of criticism refers to the fact that not all known MHC class I genes of the species studied here are compared, and only a small extract from the full repertoire of class I genes was chosen for comparison. This may bias the interpretation of data. In this respect, it might be useful to concentrate on one or two species, e.g. the 'class I-rich species, mouse, rat, or rhesus monkey. In its current form, the paper contains data from a single mouse haplotype, but from several rat haplotypes. Thus, the data set should also be updated to allow the study of both inter- and intralocus gene conversion.

**Author response: ***One of the main challenges we faced when we started to do the work that would allow us to write this paper was not in terms of "How many sequences for MHC class I molecules can we collect and align ?". It was, in fact, exactly the reverse, i.e. :" With how few sequences can we proceed to demonstrate, beyond reasonable doubt, that MHC class I loci do undergo concerted evolution ?" All the figures presented in the paper were obtained from the one alignment we settled for in the end. Changing just one sequence in the list would require performing the whole study all over again, which would represent several weeks of tenuous work*.

*Although our observations lead us to discuss many aspects related to MHC evolution, evaluating the frequency of inter and intra-locus conversion events was not within the remit of this study. Regarding the choice of MHC sequences from several rat MHC haplotypes, and from a single mouse haplotype, we do not see why this should have any relevance to the type of work we have done, and to the conclusions we reach. Outside of particular situation where genes can co-evolve because they are closely linked (such as RT1-A and TAP), MHC haplotypes are, after all, relatively artificial sets of genes that happened to find themselves on the same chromosomal strand when inbred strains were generated*.

Starting with many more sequences, we would have faced the following problems:

*1) The alignment showed on *Figure 2, *which was used to generate the trees, could not have been provided within the manuscript. We also find that the clarity of figures containing trees degrades rapidly when these trees have too many branches*.

*2) The computer time required for calculation of the trees and of the ds/dn values grows exponentially with the number of sequences, and the time spent generating the alignments and the figures is also dependent on the number of sequences included*.

*3) For the precise question we wanted to address, the only class Ib loci that were informative were those identified in at least two species. We also felt that it was best to restrict our analysis to those molecules for which a function had clearly been documented, and which had the same number of amino-acids as class Ia sequences (to avoid gaps in the alignment). When we embarked on this work, the only class Ib loci fulfilling these criteria were the CD94L and the murine M3 molecules. As far as we know, this is still true today*.

page 16: the sister grouping of M3 and CD94L genes is due to a limited data set (see above) and does not reflect true phylogenetic relationship (and is not supported by bootstrapping). Furthermore, it contradicts data by Hurt et al. (2004) who studied the phylogenetic relationship of all rat and mouse class I genes;

**Author response: ***We are in complete agreement with the statement that the grouping of M3 and CD94L does not necessarily reflect phylogenetic relationship, and this despite a bootstrapping value of 63% (with the methods used for these comparisons, values above 60% are usually considered significant, and this is specified several times in the paper). Two alternative interpretations relating to this were (and still are) proposed in the manuscript. We actually pointed to this feature of the tree to underline our point of view that extreme caution must be exerted when carrying out phylogenetic analyses of members of multigene families that undergo extensive inter-genic exchanges*.

page 16 and more: it is not obvious why the authors introduce a new abbreviation for 'residues outside the antigen recognition site (ROARS)' and do not use the widely accepted 'non-PBR';

**Author response: ***We chose to use ROARS because we think it sounds better than 'non-PBR', and also because not all class I molecules present peptides*.

page 17, second paragraph: the authors should explain how homogenisation can be afforded in non-PBRs, particularly at those sites where PBRs and non-PBRs alternate. Is the degree of homology between the two sequences high enough to allow gene conversion to take place?

**Author response: ***What we witness here are signs that are very evocative of intra-species homogenisation, and gene conversion seems to be the most likely mechanism to explain this. We have no way of knowing when these events took place, and between what sequences (for example, some other genes, or pseudogenes, could have served as relay between certain sequences). Furthermore, although gene conversion is clearly favoured between homologous sequences, we are not aware of data documenting the minimal length of homologous sequences required for gene conversion to take place. Outside of the fact that this question seems to be way beyond the scope of our study, we therefore would have no way of addressing this question*.

I would not recommend adding of the additional section into the manuscript, as the manuscript might become 'unreadable'. However, certain aspects of this "additional" discussion section might be included in the manuscript. Nevertheless, I would strongly recommend considerable shortening of the manuscript.

In my opinion, this paper should be published, but should be regarded as a 'hypothesis paper' as it contains many assumptions, which were not proven by experimental evidence, and it contains many review-like sections.

**Author response: ***As explained above, we have managed to comply to these slightly contradictory recommendations (i.e. including more points but shortening overall) by splitting the paper in two: One 'real' paper with the results, and one hypothesis paper*.

### Reviewer's report 2

#### Pierre Pontarotti

This article hypothesizes that the Peptide Binding Region of the mouse, rat and human Class I b, that presents the leader peptide from the Class I a molecules to natural killer cells, evolved from a common ancestor while the non PBR part evolved via gene conversion.

The arguments are based upon phylogenic analysis and upon the conserved location of these MHC class I b genes.

This contrasts with another hypothesis: the MHC class I genes are lineage specific, they come from a common ancestor which is different in the human and mouse lineage, (in other word class I gene from mouse and human are paralogues), and HLA E and H2Qa1 PBR evolved via convergent evolution...

In order to strengthen their hypothesis the authors should screen other mammalian lineages using ensembl data bases since some sequences of ensembl data base are not obligatory present in NCBI NR, especially those from canis, loxodanta, bos Taurus, canis Familirais and monodelphis (even if this species is out side of the eutherian group). If an "HLAE like" PBR orthologue is found in all these groups, the author hypothesis will be stronger supported.

**Author response: ***We would indeed have been very interested to identify MHC class I molecules with CD94L-like PBR outside of the rodent and primate genera. As was already indicated in the result section entitled "Certain residues are CD94L-specific, and others are homogenised within species" (on page 10 of the current manuscript), we have repeatedly tried to identify such molecules via several approaches in all the online databases available to us, and, as of 20 Dec 2005, we have not succeeded so far*.

Second if the conversion of the non PBR HLA E like gene is an ongoing process, this could be seen at higher taxonomic level, for example at primate level by comparing human chimp and macaque MHC class I genes: more homogenization should be seen outside the "HLA E like" PBR than within the PBR.

**Author response: ***This is indeed exactly what we see, and this is discussed on page 18 of the manuscript (Comparison of this tree. low support values)*

Other comments

Concerning the sentence page 14 L 11: Among the classI B ...years ago. I do not understand why the results confirm that CD94L molecules are much more evolutionary conserved than Class I a molecules.

**Author response: ***This was indeed confusing, and I have tried to clarify this point by writing the following sentence:*

*"The fact that the comparison of primate sequences strongly suggests that the four CD94L are orthologues, whereas this is much less clear for the corresponding class Ia sequences confirms previous reports that primate..."*.

## Supplementary Material

Additional File 1 *Comparison of primate α3 domains suggests past gene conversions rather than whole exon exchange*. This figure is reproduced from Figure 1 in [38]. Areas boxed in red indicate zones where the HLA-E sequence matches better with HLA-Cw1, and in green with HLA-A3. Over the zones marked in blue, all six sequences are so homologous to one another that no grouping is suggested. Click here for file
