# Supplementary material for: The orthology of HLA-E and H2-Qa1 is hidden by their concerted evolution with other MHC class I molecules
Source: Biol Direct. 2006 Jan 31;1:2. doi: 10.1186/1745-6150-1-2 (PMC1403747; doi:10.1186/1745-6150-1-2)
Supplement: Additional File 1 — Comparison of primate α3 domains suggests past gene conversions rather than whole exon exchange. This figure is reproduced from Figure 1 in [38]. Areas boxed in red indicate zones where the HLA-E sequence matches better with HLA-Cw1, and in green with HLA-A3. Over the zones marked in blue, all six sequences are so homologous to one another that no grouping is suggested. [file 1745-6150-1-2-S1.pdf]

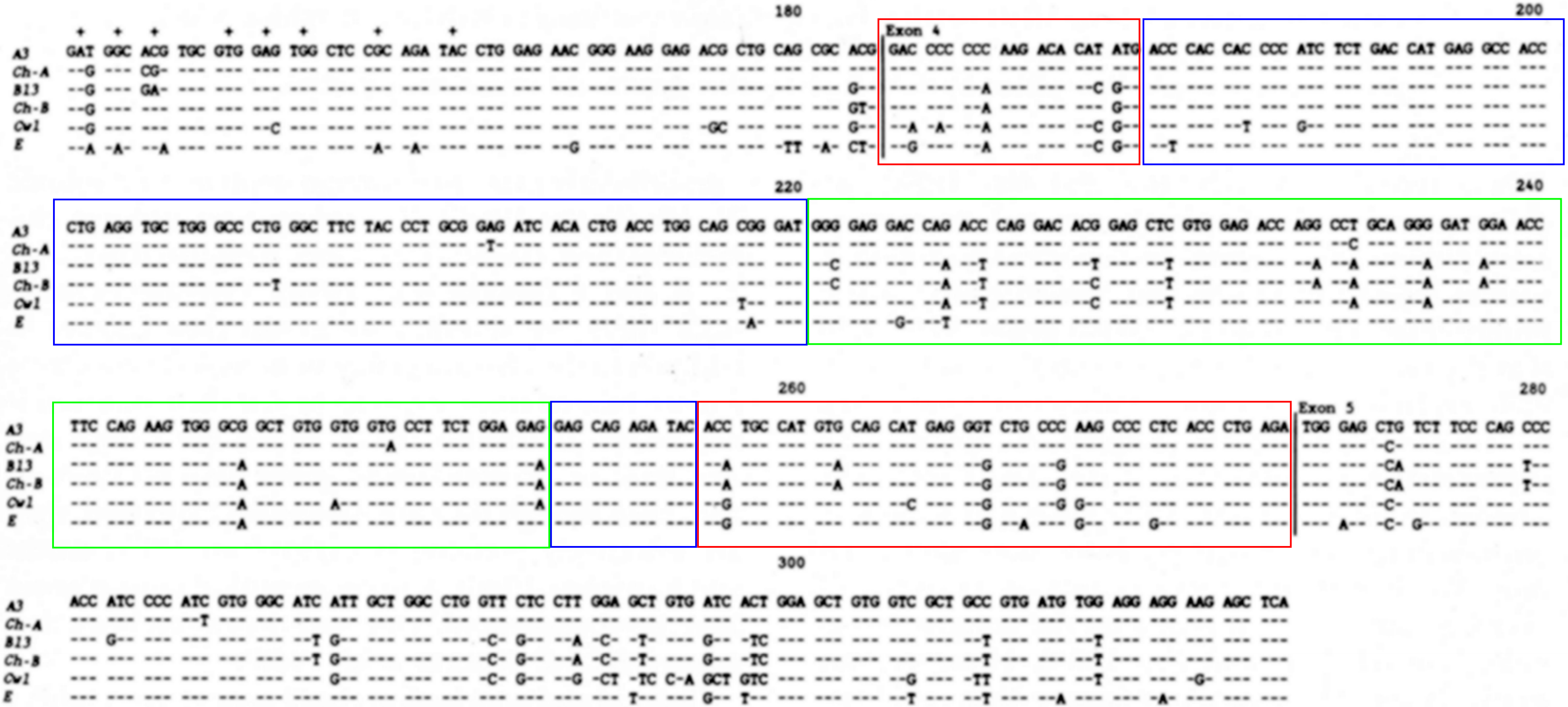

FIGURE 1.—Six DNA sequences of exons 2–5 from human and chimpanzee class I MHC loci. *A3*, *B13* and *Cw1* are human classical genes. *Ch-A* and *Ch-B* stand for the chimpanzee classical genes *CH-A108* and *CH-B2*, respectively, whereas *E* is a human nonclassical gene. Codons encoding residues in the antigen recognition site (ARS) are marked above with "+." "-" indicates identity with the *A3* sequence.
